# Clinical and chest CT presentations from 27 patients with COVID-19 pneumonia in Mogadishu, Somalia: a descriptive study

**DOI:** 10.1186/s43055-020-00302-2

**Published:** 2020-09-11

**Authors:** Yahye Garad Mohamed, Mohamed Farah Yusuf Mohamud, M. Sabri Medişoğlu, Ihsan Yavuz Atamaca, Ibrahim Hussein Ali

**Affiliations:** 1 Mogadishu Somali Turkish Training and Research Hospital, Mogadishu, Somalia; 2 Mogadishu Somali Turkish Training and Research Hospital, 30 Street, Alikamin, Wartanabada District, Mogadishu, Somalia

**Keywords:** Coronavirus disease 2019, Chest CT, Pneumonia, RT-PCR

## Abstract

**Background:**

Coronavirus disease 2019 (COVID-19) is an acute viral pneumonia that had recently been found in humans. The first case was discovered in Wuhan, Hubei province, China, in December 2019. In this article, we aimed to demonstrate the clinical and radiological characteristics of COVID-19 patients in Somalia from 20 March 2020 to 20 April 2020.

**Results:**

Twenty-seven patients that had a positive RT-PCR test between 20 March 2020 and 20 April 2020 were retrospectively observed. This study included 19 (70.4%) males and 8 (29.6%) females, and the mean age and range were 43 years (SD ± 14.0) and 27–70 years, respectively. The majority (59.3%) of COVID-19-infected patients had no obvious history of exposure to infected patients. The participants of our study mostly presented with dry cough 24 (88.9%) patients, fever 19 (70.4%), myalgia 18 (66.6%), and sore throat 16 (59.3%). Twenty-five of 27 patients had abnormal chest CT, while 2 (7.4%) patients had normal chest CT. The most common patterns of abnormality seen on chest CT in patients with COVID-19 were ground-glass opacity (GGO) 74.1%, crazy paving pattern 18.5%, consolidation 14.8%, and mixed GCO 11.1%. Also, the most common predominant lesion distributions were bilateral lung involvement (88.9%), peripheral distribution (77.8%), and lower lung predominance (63%). Particularly, lung cavitation, discrete pulmonary nodules, pleural effusion, and underlying pulmonary fibrosis or emphysema had not been observed.

**Conclusion:**

Dry cough, fever, myalgia, and sore throat were the most clinical presentations. GGO, crazy paving pattern, patchy consolidation, and mixed GCO were the typical chest CT manifestations.

## Background

Coronavirus disease 2019 (COVID-19) is an acute viral pneumonia that was recently found in humans. The first case was discovered in Wuhan, Hubei province, China, in December 2019. COVID-19 is described by the World Health Organization (WHO) as an international public health emergency of public concern and also declared COVID-19 as a pandemic [1–3].

Chen et al. demonstrated that history of exposure, clinical features, results of lab tests, and chest computed tomography (CT) findings were the basic diagnostic parameters of COVID-19 [4]. Fever, cough, shortness of breath (SOB), fatigue, and myalgia were the common clinical manifestations of COVID-19 patients [5]. As recently reported, chest CT indicates typical radiographic features in almost all COVID-19 patients, consisting of ground-glass opacities (GGOs), bilateral pulmonary consolidations, and/or interstitial changes with a peripheral distribution [6]. In this article, we report the clinical and radiological findings of twenty-seven patients with confirmed COVID-19 pneumonia in Somalia.

## Methods

The medical ethics committee approval letter of this retrospective study was received from the institutional review board of the hospital, and written patient informed consent was waived.

### Patients

From March 20 to April 20, 2020, a clinical and chest CT imaging data of 27 patients (mean age, 43 ± 14 years; 70.4% male and 29.6% female) with laboratory-confirmed reverse transcriptase polymerase chain reaction (RT-PCR) assay with throat swab samples diagnosed as SARS-CoV-2 pneumonia were included in this study.

The throat swab samples were collected for RT-PCR in accordance with the WHO guidelines [7]. The diagnosis of COVID-19 was confirmed when RT-PCR test results were positive. We excluded all the patients with negative severe acute respiratory syndrome coronavirus 2 (SARS-CoV-2) test results, pregnant women, and children (< 18 years) from this study.

All demographic characteristics, clinical signs and symptoms, laboratory results, and chest CT imaging data were retrospectively reviewed from the patient’s electronic medical records in Mogadishu Somalia Turkey Training and Research Hospital and De Martino Hospital information system (HIS)**.**

The clinical data analyzed were as follows: age, sex, comorbid conditions, exposure history, signs and symptoms (fever, dry cough, SOB, myalgia, sore throat, headache, diarrhea, vomit, and nausea), and laboratory results (C-reactive protein, leukocytes, lymphocytes, and neutrophils).

### Imaging technique and interpretation

All the patients in this study underwent non-enhanced chest CT examinations for detecting SARS-CoV-2 pneumonia in the supine position during end-inspiration.

CT data was obtained using a 16-slice configuration CT scanner (SOMATOM Emotion Siemens Healthineers Germany). The protocols were as follows: tube voltage, 120 kVp; automatic tube current modulation, 30–70 mA; and rotation time, 0.75 s. All images were then reconstructed with a slide thickness of 0.75–1.50 mm

Two radiologists (with 12 and 10 years of experience in thoracic CT) evaluated all imaging features blindly and independently in consensus.

The epidemiological history, clinical signs and symptoms (fever, dry cough, shortness of breath, and myalgia or fatigue), and laboratory tests were available for both radiologists.

We described three distributions: lung region, craniocaudal, and transverse. The main CT features included ground-glass opacities (GGO), mixed GGO, consolidation, crazy paving, air bronchogram, reticular pattern, subpleural linear opacity, bronchial dilatation, cavitation, intrathoracic lymph node enlargement, and pleural effusions. All terms were defined in accordance with the Fleischer Society guidelines [8].

### Statistical analysis

Statistical software SPSS (version 23.0, IBM) was used for all statistical analyses. Categorical variables were displayed as counts and percentages, and continuous variables were reported as mean ± standard deviation and range.

## Results

Clinical, laboratory, and radiological data of 27 patients performed oropharyngeal swab tests and confirmed as 2019-nCoV were included in this study. Epidemiologically, there were two main groups of patients: 11 (40.7%) were healthcare workers who had close contact with patients at the hospital who had confirmed COVID-19 pneumonia and the remaining 16 (59.3%) did not have any obvious history of exposure.

There were 19 (70.4%) males and 8 (29.6%) females studied; the mean age of patients was 43 ± 14.0 ranging from 27 to 70 years (Table 1). Comorbidities were present in 6 (22.2%) patients, with diabetes being the most common comorbidity, followed by hypertension and renal failure. Only one chronic obstructive pulmonary disease (COPD) patient was identified (Table 1).

**Table 1:** Clinical characteristics and laboratory results patients with SARS-CoV-2 pneumonia

Patient characteristics	Patients (*n* = 27)
Patient demographics	
Male	19 (70.4%)
Female	8 (29.6%)
Age (years), mean ± SD (range)	43 ± 14.0 (27–70)
Exposure history	
Exposure to positive patients	11 (40.7%)
Unknown exposure	16 (59.3%)
Comorbid conditions	
Diabetes	6 (22.2%)
Hypertension	3 (11.1%)
Renal failure	3 (11.1%)
COPD	1 (3.7%)
Liver disease	1 (3.7%)
Signs and symptoms	
No obvious symptoms	4 (14.8%)
Fever	19 (70.4%)
Dry cough	24 (88.9%)
SOB	6 (22.2%)
Myalgia	18 (66.6%)
Sore throat	16 (59.3%)
Headache	5 (18.5%)
Diarrhea	4 (14.8%)
Vomit	4 (14.8%)
Nausea	2 (7.4%)
Laboratory test	
C-reactive protein (mg/L; normal range 0–10)	
Increased	21 (77.8%)
Decreased	0 (0%)
Normal	4 (14.8%)
Leukocytes (normal range 4–10 × 10^9^/L)	
Increased	2 (7.4%)
Decreased	4 (14.8%)
Normal	21 (77.8%)
Lymphocytes (normal range 20–40%)	
Increased	0 (0%)
Decreased	21 (77.8%)
Normal	6 (22.2%)
Neutrophils (normal range 50–70%)	
Increased	19 (70.4%)
Decreased	0 (0%)
Normal	8 (29.6%)

The clinical features and laboratory findings of our patients are summarized in Table 1. The most common symptoms at onset were dry cough 24 (88.9%) patients, fever 19 (70.4%), myalgia 18 (66.6%), and sore throat 16 (59.3%). Other non-specific symptoms included headache 5 (18.5%) patients, diarrhea 4 (14.8%), vomiting 4 (14.8%), and nausea 2 (7.4%), whereas 4 (14.8%) patients had no obvious symptoms.

In the laboratory results, leukocytes of 21 (77.8%) patients were in the normal range, while in 4 (14.8%) patients below the normal range and in 2 (7.4%) patients above the normal range. Lymphocytes were below the normal range in most of the patients 21 (77.8%), while 19 (70.4%) patients had neutrophils above the normal range. Patients with increased CRP were more than those within the normal range (Table 1).

**Fig. 1:**
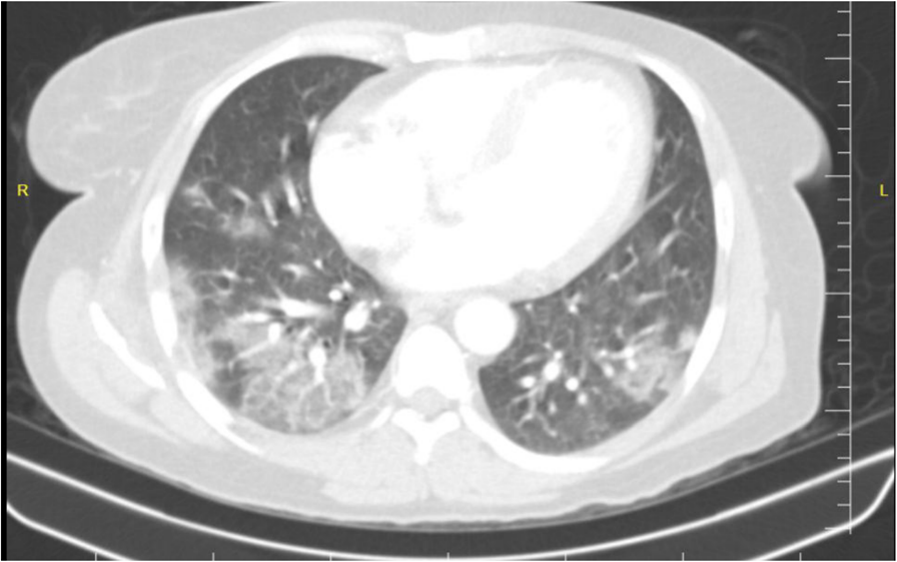
An axial chest CT from a 47-year-old woman with COVİD-19 pneumonia. Non-contrast-enhanced chest CT image showing multiple peripheral ground-glass opacities distributed in the bilateral multiple lobular and subsegmental areas

**Fig. 2:**
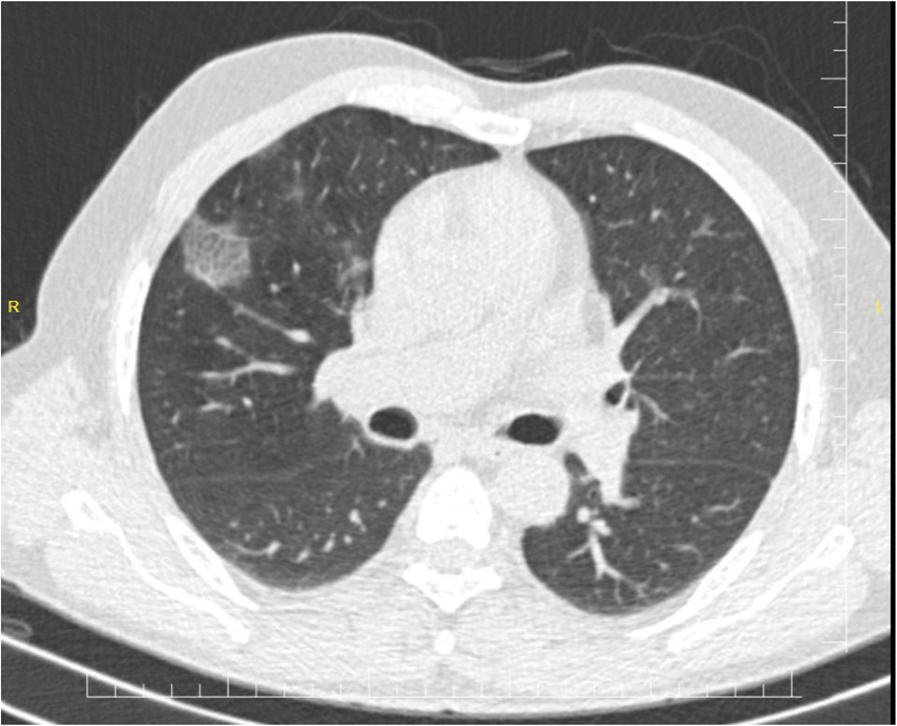
An axial chest CT from a 50-year-old man with COVİD-19 pneumonia. Non-contrast-enhanced chest CT image showed focal ground-glass opacity associated with smooth interlobular and intralobular septal thickening in the right upper lobe. The left lung was normal

**Fig. 3:**
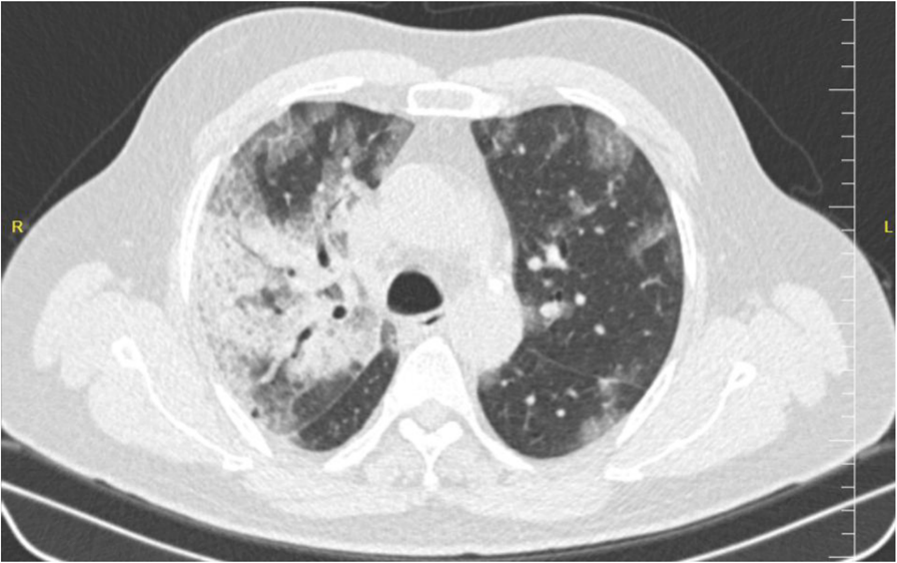
An axial chest CT from a 60-year-old man with COVİD-19 pneumonia. Non-contrast-enhanced chest CT image showed multiple peripheral ground-glass opacities distributed in the bilateral lung fields, combined with consolidation

**Fig. 4:**
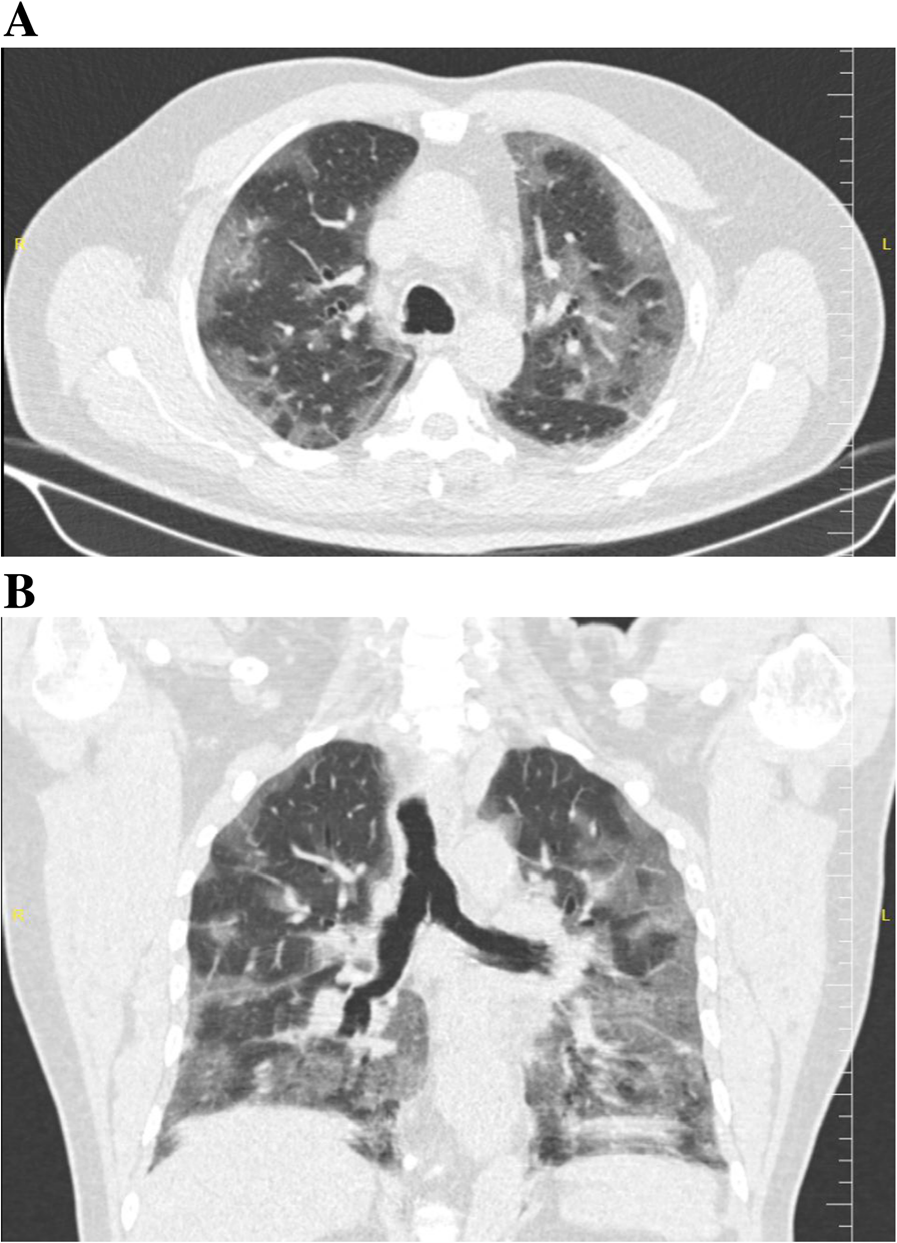
Axial unenhanced chest CT image (**a**) and coronal reconstructed unenhanced chest CT image (**b**) from a 57-year-old man with COVİD-19 pneumonia noted widespread ground-glass and consolidative opacities in both lungs with a bilateral, peripheral, and basal predominant distribution

**Fig. 5:**
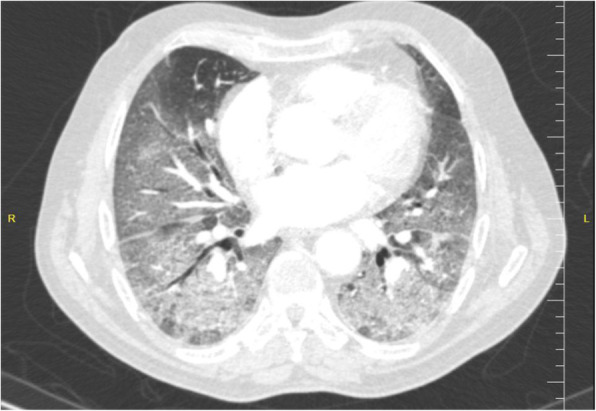
An axial chest CT from a 60-year-old man with COVİD-19 pneumonia. Non-contrast-enhanced chest CT image demonstrated extensive ground-glass opacities seen in both lungs, involving almost all lower lobes, giving a white lung appearance, with air bronchograms

As shown in Table 2, 25 (92.6%) of 27patients had abnormal chest CT, while 2 (7.4%) patients had normal chest CT. The most common patterns seen on chest CT in patients with COVID-19 were GGO 20 (74.1%) patients (Fig. 1), crazy paving pattern 5 (18.5%) patients (Fig. 2), patchy consolidation 4 (14.8%) patients, and mixed GCO 3 (11.1%) patients (Fig. 3). The most common predominant lesion distributions were bilateral lung involvement (88.9%), peripheral distribution (77.8%), and lower lung predominance (63%) (Fig. 4a, b). Also, air bronchogram (Fig. 5) and lymphadenopathy were also noted (Table 2). Particularly, lung cavitation, discrete pulmonary nodules, pleural effusions, and underlying pulmonary fibrosis or emphysema were not observed.

**Table 2:** Imaging findings of patients with SARS-CoV-2 at presentation

Chest CT finding	Patients (*n* = 27)
Patterns of the lesion	
Ground-glass opacification	20 (74.1%)
Mixed ground-glass opacities and consolidation	3 (11.1%)
Consolidation	4 (14.8%)
Crazy paving pattern	5 (18.5%)
Air bronchogram sign	2 (7.4%)
Reticular pattern	1 (3.7%)
Subpleural linear opacity	2 (7.4%)
Bronchial dilatation	2 (7.4%)
Lymphadenopathy	1 (3.7%)
Cavitation	0 (0%)
Pleural effusion	0 (0%)
Distribution	
Craniocaudal distribution	
Upper lung predominance	1 (3.7%)
Lower lung predominance	17 (63%)
No craniocaudal distribution	9 (33.3%)
Transverse distribution	
Central	1 (3.7%)
Peripheral	21 (77.8%)
No transverse distribution	6 (22.2%)
Lung region distribution	
Unilateral	1 (3.7%)
Bilateral	24 (88.9%)
No lung region distribution	2 (7.4%)

## Discussion

In this study, we investigated the clinical characteristics and radiographic features of COVID-19 pneumonia in adults, besides those from pregnant women. Our study found that COVID-19 patients had a variety of clinical and radiological manifestations. Cough, fever, myalgia, and sore throat were the most common clinical manifestations in our study similar to previous studies [4, 5]. Other non-specific symptoms included SOB, diarrhea, vomiting, nausea, and headache which occurred in around 7–18% of patients.

Chest CT is an essential part of the diagnostic investigation for patients with suspected COVID-19 infection, although very few cases have normal chest CT findings at the early stage [9]. However, our study has shown variants of chest CT findings in affected patients. Twenty-three of 27 (85.1%) symptomatic patients had abnormal chest CT, while 2 (7.4%) of 4 (14.8%) asymptomatic patients had normal chest CT and none of them had any comorbid disease. Based on recently published data, most of the patients with COVID-19 had characteristics of chest CT features in the disease process such as ground-glass opacities (GGOs), mixed GGC, crazy paving pattern, consolidation, air bronchogram, bronchial dilatation, reticular pattern, and subpleural linear opacity [10–12].

In the present study, the most commonly observed opacification in patients with COVID-19 was GGO in 20 (74.1%), which appeared predominantly in the peripheral zones and most often involved lower lung lobes and segments which are consistent with the results of previous studies of COVID-19 and other viruses including SARS and MERS [13–15]. Furthermore, the most common characteristics of chest CT features associated with COVID-19 pneumonia in the elder group (> 60 years) included GGO with consolidation or reticular pattern, and consolidation with predominantly peripheral distribution and bilateral lung involvement, which was similar to the previous studies [16, 17].

In our study, we had several limitations; first, it was a retrospective study, and we had a comparatively small number of 27 patients. On the one hand, we only analyzed the clinical and chest CT manifestations at the initial presentation. Further studies are needed including clinical and radiological follow-ups.

Second, not all laboratory tests were done in all patients, including procalcitonin, AST, ALT, creatinine, urea, albumin, procalcitonin, d-dimer, lactate dehydrogenase, and serum ferritin.

Finally, the article only included non-pregnant adults. The clinical characteristics and radiological findings of pregnant women and children infected with COVID-19 are not clear.

## Conclusion

This study represents an early investigation of clinical characteristics and chest CT findings in patients with 2019 novel coronavirus (2019-nCoV). Dry cough, fever, myalgia, and sore throat were the most clinical presentations.

GGO, crazy paving pattern, patchy consolidation, and mixed GCO were typical chest CT manifestations. The most common predominant lesion distributions were bilateral, peripheral and lower lung zone predominance. A small number of patients with COVID-19 pneumonia had had no obvious symptoms, and there chest CT scans were normal.

## Data Availability

The data that support the findings of this study are available from Mogadishu Somali Turkish Training and Research Hospital. Data are however available from the authors upon reasonable request and with permission from Mogadishu Somali Turkish Training and Research Hospital.

## References

[1] Huang C, Wang Y, Li X, Ren L, Zhao J, Hu Y, Zhang L, Fan G, Xu J, Gu X, Cheng Z (2020). Clinical features of patients infected with 2019 novel coronavirus in Wuhan, China. Lancet..

[2] Zhu N, Zhang D, Wang W, Li X, Yang B, Song J, Zhao X, Huang B, Shi W, Lu R, Niu P A novel coronavirus from patients with pneumonia in China, 2019, New England Journal of Medicine. 2020 10.1056/NEJMoa2001017 PMC7092803 31978945

[3] WHO Director-General’s opening remarks at the media briefing on COVID 19 https://www.who.int/dg/speeches/detail/who-director-general-s-opening-remarks-at-the-media-briefingon-covid-19%2D%2D-11-march-2020.

[4] Chen N, Zhou M, Dong X, Qu J, Gong F, Han Y, Qiu Y, Wang J, Liu Y, Wei Y, Yu T (2020). Epidemiological and clinical characteristics of 99 cases of 2019 novel coronavirus pneumonia in Wuhan, China: a descriptive study. Lancet.

[5] Huang C (2020). Clinical features of patients infected with 2019 novel coronavirus in Wuhan, China. Lancet.

[6] Chung M, Bernheim A, Mei X, Zhang N, Huang M, Zeng X, Cui J, Xu W, Yang Y, Fayad ZA, Jacobi A (2020). CT imaging features of the 2019 novel coronavirus (2019-nCoV). Radiology..

[7] Chavez S, Long B, Koyfman A, Liang SY (2020) Coronavirus disease (COVID-19): a primer for emergency physicians. Am J Emerg Med 10.1016/j.ajem.2020.03.036 PMC7102516 32265065

[8] Hansell DM, Bankier AA, MacMahon H, McLoud TC, Muller NL, Remy J (2008). Fleischner Society: glossary of terms for thoracic imaging. Radiology..

[9] Kanne JP, Little BP, Chung JH, Elicker BM, Ketai LH (2020) Essentials for radiologists on COVID-19: an update-radiology scientific expert panel [published online ahead of print, 2020 Feb 27]. Radiology.:200527 10.1148/radiol.2020200527 PMC7233379 32105562

[10] Lei J, Li J, Li X et al (2020) CT imaging of the 2019 novel coronavirus (2019-nCoV) pneumonia. Radiology. 10.1148/radiol.2020200236 10.1148/radiol.2020200236 PMC7194019 32003646

[11] Huang P, Liu T, Huang L et al (2020) Use of chest CT in combination with negative RT-PCR assay for the 2019 novel coronavirus but high clinical suspicion. Radiology. 10.1148/radiol.2020200330 10.1148/radiol.2020200330 PMC7233360 32049600

[12] Cheng Z et al (2020) Clinical features and chest CT manifestations of coronavirus disease 2019 (COVID-19) in a single-center study in Shanghai, China. Am J Roentgenol:1–6 10.2214/AJR.20.22959 32174128

[13] Zhao W, Zhong Z, Xie X, Yu Q, Liu J (2020). Relation between chest CT findings and clinical conditions of coronavirus disease (COVID-19) pneumonia: a multicenter study. Am J Roentgenol.

[14] Wong KT, Antonio GE, Hui DS, Lee N, Yuen EH, Wu A, Leung CB, Rainer TH, Cameron P, Chung SS, Sung JJ (2003). Thin-section CT of severe acute respiratory syndrome: evaluation of 73 patients exposed to or with the disease. Radiology..

[15] Gao F, Li M, Ge X, Zheng X, Ren Q, Chen Y, Lv F, Hua Y (2013). Multidetector spiral CT study of the relationships between pulmonary ground-glass nodules and blood vessels. Eur Radiol.

[16] Chung M, Bernheim A, Mei X, Zhang N, Huang M, Zeng X, Cui J, Xu W, Yang Y, Fayad ZA, Jacobi A (2020). CT imaging features of 2019 novel coronavirus (2019-nCoV). Radiology..

[17] Song F, Shi N, Shan F, Zhang Z, Shen J, Lu H, Ling Y, Jiang Y, Shi Y (2020). Emerging 2019 novel coronavirus (2019-nCoV) pneumonia. Radiology..

